# Bacterial pathogens in pediatric appendicitis: a comprehensive retrospective study

**DOI:** 10.3389/fcimb.2023.1027769

**Published:** 2023-05-09

**Authors:** Julia Felber, Benedikt Gross, Arend Rahrisch, Eric Waltersbacher, Evelyn Trips, Percy Schröttner, Guido Fitze, Jurek Schultz

**Affiliations:** ^1^ Department of Pediatric Surgery, University Hospital Dresden – Technical University of Dresden, Dresden, Germany; ^2^ Coordination Centre for Clinical Trials, Faculty of Medicine Carl Gustav Carus, Technical University of Dresden, Dresden, Germany; ^3^ Institute for Microbiology and Virology, University Hospital Dresden – Technical University of Dresden, Dresden, Germany

**Keywords:** pediatric appendicitis, rare bacteria, anti-infective treatment, complications, outcome

## Abstract

**Background:**

Appendicitis is a frequent condition, with peak incidences in the second decade of life. Its pathogenesis is under debate, but bacterial infections are crucial, and antibiotic treatment remains essential. Rare bacteria are accused of causing complications, and various calculated antibiotics are propagated, yet there is no comprehensive microbiological analysis of pediatric appendicitis. Here we review different pre-analytic pathways, identify rare and common bacterial pathogens and their antibiotic resistances, correlate clinical courses, and evaluate standard calculated antibiotics in a large pediatric cohort.

**Method:**

We reviewed 579 patient records and microbiological results of intraoperative swabs in standard Amies agar media or fluid samples after appendectomies for appendicitis between May 2011 and April 2019. Bacteria were cultured and identified *via* VITEK 2 or MALDI-TOF MS. Minimal inhibitory concentrations were reevaluated according to EUCAST 2022. Results were correlated to clinical courses.

**Results:**

Of 579 analyzed patients, in 372 patients we got 1330 bacterial growths with resistograms. 1259 times, bacteria could be identified to species level. 102 different bacteria could be cultivated. 49% of catarrhal and 52% of phlegmonous appendices resulted in bacterial growth. In gangrenous appendicitis, only 38% remained sterile, while this number reduced to 4% after perforation. Many fluid samples remained sterile even when unsterile swabs had been taken simultaneously. 40 common enteral genera were responsible for 76.5% of bacterial identifications in 96.8% of patients. However, 69 rare bacteria were found in 187 patients without specifically elevated risk for complications.

**Conclusion:**

Amies agar gel swabs performed superior to fluid samples and should be a standard in appendectomies. Even catarrhal appendices were only sterile in 51%, which is interesting in view of a possible viral cause. According to our resistograms, the best *in vitro* antibiotic was imipenem with 88.4% susceptible strains, followed by piperacillin-tazobactam, cefuroxime with metronidazole, and ampicillin-sulbactam to which only 21.6% of bacteria were susceptible. Bacterial growths and higher resistances correlate to an elevated risk of complications. Rare bacteria are found in many patients, but there is no specific consequence regarding antibiotic susceptibility, clinical course, or complications. Prospective, comprehensive studies are needed to further elicit pediatric appendicitis microbiology and antibiotic treatment.

## Background

Appendicitis is among the most frequently treated surgical conditions, with peak incidences in the second decade of life (Bhangu et al., 2015). The disease occurs with a global incidence of 100 per 100.000 people while reaching even 151 per 100.000 in Germany (Körner et al., 1997). Due to its specifically high incidence from 10 to 14 years in boys and 10 to 19 years in girls (Andersen et al., 2009; Ohmann et al., 2014; Jaya Kumar et al., 2017), there is a need for a detailed analysis of this disease in a pediatric cohort.

For many decades, bacterial transmigration and invasive infections were thought to be critical in the development and progression of appendicitis. Consequently, perioperatively administered antibiotic prophylaxis and treatment remain essential in its management. Thus, different calculated antibiotics and antibiotic combinations have been discussed in the past, and still, different guidelines exist on this matter. Most importantly, no specific up-to-date guidelines on pediatric appendicitis exist, which makes an analysis of bacterial pathogens and their antibiotic resistance in a pediatric cohort even more valuable. Therefore, in this study, we test four different commonly used antibiotic agents as they have been advocated in the past and used in our department: ampicillin-sulbactam (Kambaroudis et al., 2010; Kronman et al., 2016), cefuroxime with metronidazole (Sauerland et al., 2010; Rollins et al., 2016), piperacillin-tazobactam (Fallon et al., 2013; Mazuski et al., 2017; Sartelli et al., 2017; Roque et al., 2019b), and imipenem (Kambaroudis et al., 2010; Mazuski et al., 2017; Sartelli et al., 2017). In addition, a comprehensive analysis of bacterial growths in pediatric appendicitis is fundamental, given the recent debate on treating uncomplicated appendicitis conservatively.

Recently many authors propagated antibiotic management without initial surgery for uncomplicated appendicitis (Varadhan et al., 2012; Rollins et al., 2016), and various evidence points to a different pathomechanism for uncomplicated and complicated appendicitis (Livingston et al., 2007; Rawolle et al., 2019). For the former, a viral cause is debated (Andersson et al., 1995; Alder et al., 2010; Richardsen et al., 2016), which might leave transmural migration of bacteria to gangrenous and perforated appendicitis. Because of these arguments, the microbiological analysis of catarrhal and phlegmonous appendicitis is also intriguing.

Studies have advocated blood culture bottles for sample collection (Jiménez et al., 2019) when others propagate routine swabs (Davies et al., 2010; Son et al., 2020). Given the importance of good coverage of possibly causative bacteria in appendicitis, comparing different modes of sample collection will add to the discussion.

Some rare bacteria have been accused of causing complications in appendicitis: peritonitis caused by *Actinomyces odontolyticus* (Lopes et al., 2017), suspected bowel perforation (Legaria et al., 2020) and abscess formation by *Clostridium ramosum* (Forrester and Spain, 2014) and *Eikenella corrodens* (Paul and Patel, 2001), free abdominal fluid (in perforated appendicitis) and psoas abscess by *Comamonas kerstersii* (Almuzara et al., 2013; Almuzara et al., 2017), gangrenous appendicitis by *Eggerthella lenta* (Gardiner et al., 2015). However, since case presentations usually arise from complications, the pathogenicity of rare bacteria can easily be overestimated. Therefore, a comprehensive approach might help to get a hold of uncomplicated clinical courses despite rare bacteria.

Finally, various calculated antibiotics or combinations are propagated for appendicitis, yet there is no comprehensive microbiological analysis of pediatric appendicitis. Here we review different pre-analytic pathways, identify bacteria, rare pathogens, and their resistances, correlate clinical courses, and evaluate standard calculated antibiotic managements in a large pediatric cohort.

## Materials and methods

We reviewed patient records and microbiological results of all appendectomies due to appendicitis between May 2011 and April 2019. During this period, two types of samples were sent for microbiological analysis: either an intraoperative swab was wiped by the surgeon along the serosa of the appendix and sent in a conventional Amies gel transport medium (Sarstedt AG & Co. KG, Nümbrecht, Germany) (Van Horn et al., 2008; Reinisch et al., 2017) or intraabdominal fluid was aspirated and directly sent natively to microbiology (12 ml PS Tube, sterile, greiner bio-one GmbH, Frickenhausen, Germany). All samples were directly analyzed within routine microbiological diagnostics. In 110 cases, both swabs and native material were sent.

Both, swabs and fluids, were processed according to the standard routine procedures of the microbiology laboratory (**Supplementary Figure 1**). Bacteria were identified *via* VITEK 2 or MALDI-TOF MS and minimal inhibitory concentrations were determined with routine methods and evaluated according to EUCAST 2022 (The European Committee on Antimicrobial Susceptibility Testing. Breakpoint tables for interpretation of MICs and zone diameters. Version 12.0, 2022. http://www.eucast.org.). For this, the minimal inhibitory concentrations (MIC) were individually retrieved from our laboratory reports and re-evaluated with current EUCAST breakpoints. We reviewed 619 children 2 to 17 years of age with postoperatively confirmed appendicitis. All found bacteria and resistances were evaluated and correlated to clinical courses. In addition, we estimated the specific incidence and performed literature research on each identified species to identify rare pathogenic bacteria.

### Definition of complications

When evaluating the clinical course, we defined complications as unplanned outpatient visits after appendectomy for pain, wound healing problems, or GI-symptoms. Further complications included readmissions for gastrointestinal problems and re-operations for abdominal problems within one year after appendectomy. However, when patient records revealed unusual pain, fever, delayed enteral nutrition, or constipation during initial inpatient treatment, this was noted as a complication only if inpatient treatment lasted longer than the average hospital stay of 7 days.

### Definition of rare bacterial pathogens

There is no commonly agreed definition for rare bacterial pathogens. Like in rare diseases, accepted definitions include the low number of affected patients and the little knowledge on this disease. Commonly used definitions for rare diseases are based on prevalence which works well for chronic conditions but risks omitting short-lasting illnesses. To overcome this problem, the RARECARE project chose an incidence-based definition for rare cancers as those with an annual incidence of less than six per 100,000 people (Gatta et al., 2017). For this study, we deducted our definition for rare bacterial pathogens from the above-mentioned criteria: since, in Germany, appendicitis has an overall incidence of 151/100 000 (Ferris et al., 2017), any bacteria found in less than 4% of our appendicitis patients or less than 23 of 579 cases, was considered to have a “rare incidence”.

However, to be termed a “rare pathogen”, a bacteria should have little published evidence in regard to human infections. To accomplish this discrimination, we searched MEDLINE *via* PubMed on Juli 15^th^, 2022 for the name of the bacteria AND “human” AND “infection”. For rarely published bacteria, we set an arbitrary threshold at any bacteria with less than 0.3% of publications on human infections with *E. coli*, the most common bacteria in human appendicitis (Wilms et al., 2011; Fallon et al., 2013; Kenig and Richter, 2013; Bhangu et al., 2015; Tartar et al., 2018; Son et al., 2020; Plattner et al., 2021).

### Statistics

In this exploratory analysis, continuous data were described by mean and standard deviation or median and interquartile range, as appropriate. Categorical data were presented by absolute and relative frequencies. Data observed in different groups were tested for differences by t-test for independent groups, paired t-test, chi-square test, Fisher’s exact test or McNemar test, as appropriate. When comparing more than two groups, the Kruskal-Wallis-Test was used. Multiple logistic regression was performed to investigate the influence of independent risk factors on complications.

Significance level was set to 5 percent. As the analyses focused on description and hypotheses generation, no adjustment of type-one-error for multiple testing was applied. Statistical analyses were performed by Microsoft Excel version 2016 and SAS version 9.4.

## Results

### Descriptive statistics of patients and management

**Figure 1:**
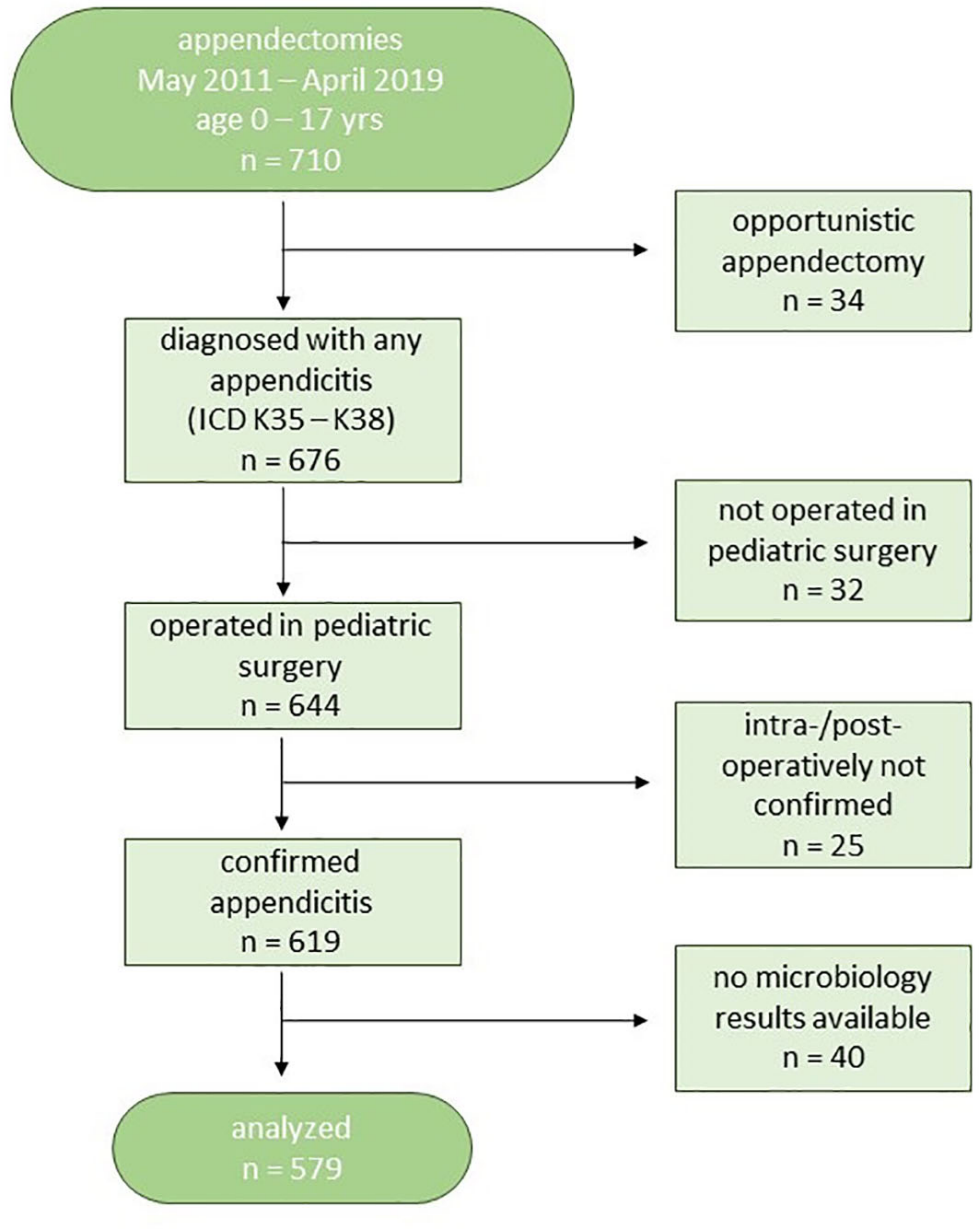
Patient flow-chart.

In the analyzed 8-year period, we screened 710 appendectomies (OPS 5-470.x). We excluded 34 patients who were opportunistically appendectomized during other operations. Another 32 patients above 16 years of age were not treated in the department of pediatric surgery and therefore excluded from further analysis. From the remaining 644 patients, we excluded 25 patients in whom no appendicitis could be confirmed intraoperatively (false positive = 3.9%). Another 40 patients had to be excluded because they did not have microbiological results in their records for various reasons, e.g., sample not taken, sample lost, no valid results due to long transportation, or irretrievable results. This left 579 patients for our analysis (**Figure 1**).

Our patients were on average 10.3 years old (range 2-17) with a ratio of 308 (53.2%) boys to 271 girls. Open appendectomy was performed in 4.8% of patients. The remaining 95.2% were operated endoscopically, either as conventional laparoscopy in three-port-technique or with single-incision or single-port technique. In 7.6% of endoscopic procedures, there was an intraoperative conversion to a laparotomy.

Intraoperatively the vermiform appendix was evaluated macroscopically by the operating surgeon. Thus we classified the appendicitis according to the operation report into simplex (n=25/644 = 3.9%, excluded from further analysis), catarrhal (n=102/579 = 17.6%), phlegmonous (n=215/579 = 37.1%), gangrenous (n=120/579 = 20.7%), and perforated (n=142/579 = 24.5%). Across all patients, the length of inpatient treatment was analyzed after excluding 6 oncological patients who stayed in the hospital due to their underlying disease. The median length of admission for the remaining 573 patients was 6 days with an interquartile range (IQR) of 5 to 10 (**Supplementary Figure 2**).

In the postoperative course, we found minor complications or adverse events in 21.2% (n=123/579). However, only 35/579 = 6% required a surgical re-intervention such as percutaneous or transrectal drainage of an abscess or re-operation. The most frequent minor complication was unusual pain (n = 32) and prolonged wound healing (n = 19) due to dehiscence or minor local infections, followed by fever and gastrointestinal symptoms such as constipation, vomitus, or diarrhea.

### Statistics of pre-analytics

An intraoperative swab was taken and sent in a conventional Amies agar gel transport medium in 85.8% of cases (n = 497). Intraabdominal fluid was sent natively to microbiology in 33.2% (n = 192). In 19.0% (110 patients), both swabs and native material were sent. In 387 patients, only a swab was taken, while in 82 cases, we only analyzed intraabdominal fluid samples. All three groups (swabs, fluid, and both) were comparable in terms of operative access, mode of ligation, the intraoperative status of the appendix, and the rate of complications (**Supplementary Table 1**).

Additionally, both swabs and fluids generated comparable top-20-profiles of identified bacteria (**Supplementary Figure 3**).

Only 156/497 = 31.4% of all swabs turned out to be sterile as no bacteria could be cultivated. This contrasts with 138/192 = 71.9% of all native fluid samples that did not result in bacterial growth in our laboratory. When excluding the patients with both swab and fluid sample, these proportions do not change to a relevant extend (**Table 1**). Even when comparing only patients with both types of samples taken simultaneously, we saw sterile results in 54/110 (49.1%) fluid samples when bacterial growth was detected from the corresponding swabs. Only 4/110 (3.6%) of fluid samples resulted in bacterial growth when the corresponding swab remained sterile. This is in good accordance with the number of identified species: on average, 2.2 species could be identified from bacterial swabs when fluid samples delivered only 1.3 different species (**Tables 1**–**3**). Around 85.7% of all identified bacteria (n = 1330) were found due to bacterial swabs when only 14.2% of identified bacteria originated in the analysis of fluid samples.

**Table 1:** Comparison of swaps and fluid samples.

Total (n = 469)	Swab only (n = 387)		Fluid only (n = 82)	
sum of identified bacteria	870		103	
samples with bacterial growth	264	68.2%	27	32.9%
samples that remained sterile	123	31.8%	55	67.1%
mean value of species identified	2.24		1.25	
SD of species identified"	2.24		2.38	
variance of species identified	5.04		5.70	
range of species identified	13		15	
p-value	<0.001			

**Table 2:** Comparison of swabs and fluid samples that were taken simultaneously.

	Simultaneuous swabs (n = 110)		Simultaneuous fluids (n = 110)	
sum of identified bacteria	270		87	
number of sterile samples	33	30.00%	83	75.45%
number of samples with bacteriel growth	77	70.00%	27	24.54%
mean value of species identified	2.45		0.79	
SD of species identified	2.34		1.60	
variance of species identified	5.52		2.56	
p-value (paired t-test)	<0.001			

**Table 3:** Cross-table of simultaneously taken samples.

Total (n = 110)	Sterile swab	Swab with bacteria	Total
sterile fluid	29	54	83
fluid with bacteria	4	23	27
total	33	77	110
p-value	<0.001		

### Statistics of bacterial growths

We identified bacteria in 136/142 = 95.8% of perforated appendicitis. Even in catarrhal appendicitis, bacteria were found in 50/102 = 49% of patients. The different rates of sterile results in the four forms of appendicitis were statistically significant (p<0.001) (**Table 4**). We only had 25 false positives without signs of inflammation. In these patients, microbiological material was mostly not taken or other pathologies than appendicitis were present.

**Table 4:** Number and rarity of bacteria found in different forms of appendicitis.

579 patients analyzed	Catarrhal (n = 102)		Phlegmonous (n = 215)		Gangrenous (n = 120)		Perforated (n = 142)	
207 patients without bacterial growth	52	51.0%	103	47.9%	46	38.3%	6	4.2%
372 patients with bacterial growth	50	49.0%	112	52.1%	74	61.7%	136	95.8%
of these 372, patients with rare pathogens	27	7.3%	41	11.0%	33	8.9%	86	23.1%
1330 bacteria detected in total	146		322		244		618	
thereof number rare bacteria	47	32.2%	72	22.4%	54	22.1%	139	22.5%
bacteria per patient (n = 579)	1.43		1.5		2.03		4.35	
bacteria per patient (n = 372), steriles excluded	2.92		2.88		3.3		4.54	
number different species	52		69		57		81	
thereof rare species	20	38.5%	28	40.6%	24	42.1%	37	45.6%

When on average, 1.43 different species were found in all catarrhal appendicitis, we found 1.5 in phlegmonous, 2.03 in gangrenous, and 4.35 in perforated appendicitis. When excluding sterile samples from the analysis, catarrhal appendicitis delivered on average 2.92, phlegmonous 2.88, gangrenous 3.3, and perforated appendicitis delivered 4.54 different species (**Table 4**). The proportion of rare bacteria in different forms of appendicitis did not differ significantly (**Table 4**). In all stages, *E. coli* was the dominating species, followed by different members of the genus *Bacteroides* and *Pseudomonas* (**Supplementary Figures 4**–**7**)

Primarily open (10.7%) and converted (4.8%) appendectomies had by far the lowest rates of sterile results, while the rate of sterile samples was highest in laparoscopically operated patients (39.7%) (**Supplementary Table 2**).

All analyzed patients with bacterial growths had, on average, 3.58 different bacteria (range 1 to 15, SD=2.27). When excluding the sterile samples, almost 90% of patients had 6 or less different bacteria (**Supplementary Figure 8**).

### Statistics of pathogens

Since many patients had a polymicrobial spectrum, the frequency of detected bacteria does not fully correspond to the rate of patients positive for certain bacteria. The most frequently detected genus among all detected bacteria was *Bacteroides* spp. (367/1330) followed by *Escherichia* spp. (315/1330), *Streptococcus* spp. (139/1330), *Pseudomonas* spp. (75/1330), *Bilophila* spp. (54/1330), and *Enterococcus* spp. (49/1330) (**Supplementary Figure 9**).

However, *Escherichia* spp. was detected in the samples of most patients (282/579), followed by *Bacteroides* spp. (252/579), *Streptococcus* spp. (114/579), *Pseudomonas* spp. (70/579), *Bilophila* spp. (53/579), and *Enterococcus* spp. which was only present in 43/579 (**Supplementary Figure 10**).

We identified bacteria down to species level 1259 times, thus totaling 102 different bacterial species. Without surprise, *E. coli* was the most found species, followed by *B. fragilis*, *P.aeruginosa*, *S. anginosus* and *B. wadsworthia*. We evaluated all bacteria found according to our definition of rare bacterial pathogens: a specific annual incidence below 6/100000 and less than 0.3% of publications compared to the most frequently published pathogen. The bacteria with the highest incidence in human pediatric appendicitis was *E. coli*. This bacteria also generated the most hits on MEDLINE, which is 66,199. Therefore, any bacteria with less than 199 publications related to human infections were considered to have little publications. Combining both criteria, we defined rare bacterial pathogens in pediatric appendicitis (**Table 5**).

**Table 5:** Identified bacteria and associated complication rates, rarity marked with background color.

Species (n = 102)	Number of publications	Study cohort		
		Detected frequency	Complications	Complication rate
*Escherichia coli*	66199	312	74/312	23.7%
*Staphylococcus aureus*	63663	27	7/27	25.9%
*Helicobacter pylori*	35634	1	0/1	0.0%
*Pseudomonas aeruginosa*	30423	75	22/75	29.3%
*Streptococcus pneumoniae*	23255	5	1/5	20.0%
*Klebsiella pneumoniae*	14915	14	2/14	14.3%
*Haemophilus influenzae*	14351	2	1/2	50.0%
*Streptococcus pyogenes*	11029	4	1/4	25.0%
*Staphylococcus epidermidis*	7717	6	0/6	0.0%
*Enterococcus faecalis*	6434	12	3/12	25.0%
*Enterococcus faecium*	3728	7	1/7	14.3%
*Yersinia enterocolitica*	3094	1	0/1	0.0%
*Proteus mirabilis*	2997	7	5/7	71.4%
*Bacteroides fragilis*	2583	207	57/207	27.5%
*Clostridium perfringens*	2567	1	0/1	0.0%
*Stenotrophomonas maltophilia*	1587	1	1/1	100.0%
*Fusobacterium nucleatum*	1469	27	6/27	22.2%
*Streptococcus sanguinis*	1062	3	0/3	0.0%
*Klebsiella oxytoca*	923	16	3/16	18.8%
*Prevotella intermedia*	855	5	4/5	80.0%
*Citrobacter freundii*	797	10	2/10	20.0%
*Fusobacterium necrophorum*	750	4	0/4	0.0%
*Staph.haemolyticus*	733	1	0/1	0.0%
*Eikenella corrodens*	633	2	1/2	50.0%
*Streptococcus anginosus*	583	66	23/66	34.8%
*Streptococcus intermedius*	494	11	7/11	63.6%
*Streptococcus salivarius*	493	3	0/3	0.0%
*Staphylococcus lugdunensis*	485	2	0/2	0.0%
*Staphylococcus hominis*	463	2	2/2	100.0%
*Morganella morganii*	440	2	0/2	0.0%
*Streptococcus gordonii*	422	2	0/2	0.0%
*Streptococcus constellatus*	310	38	13/38	34.2%
*Staphylococcus capitis*	262	1	0/1	0.0%
*Aeromonas veronii*	253	2	1/2	50.0%
*Bacteroides thetaiotaomicron*	231	53	15/53	28.3%
*Prevotella nigrescens*	228	7	4/7	57.1%
*Bifidobacterium longum*	210	4	0/4	0.0%
*Streptococcus mitis/oralis*	202	1	0/1	0.0%
*Enterobacter cloacae*	201	4	1/4	25.0%
*Citrobacter koseri*	196	4	1/4	25.0%
*Parvimonas micra	179	12	4/12	33.3%
*Cutibacterium acnes*	167	4	1/4	25.0%
*Gemella morbillorum	157	7	3/7	42.9%
*Enterococcus gallinarum*	151	2	1/2	50.0%
*Peptostrep. anaerobius*	123	1	0/1	0.0%
*Actinomyces odontolyticus*	122	2	1/2	50.0%
*Providencia rettgeri*	118	3	1/3	33.3%
*Bacteroides vulgatus*	95	45	9/45	20.0%
*Finegoldia magna*	94	1	1/1	100.0%
*Neisseria sicca*	84	1	0/1	0.0%
*Enterococcus avium*	83	28	5/28	17.9%
*Raoultella planticola*	79	1	0/1	0.0%
*Aggregatibacter aphrophilus*	72	1	0/1	0.0%
*Granulicatella adiacens*	72	1	0/1	0.0%
*Bilophila wadsworthia	66	54	16/54	29.6%
*Listeria ivanovii*	59	1	0/1	0.0%
*Bifidobacterium adolescentis*	57	1	0/1	0.0%
*Salmon. enter. ser. Typhimurium*	48	1	0/1	0.0%
*Bacteroides ovatus	47	30	7/30	23.3%
*Eggerthella lenta	47	6	2/6	33.3%
*Bacteroides uniformis	38	11	4/11	36.4%
*Clostridium ramosum*	36	2	1/2	50.0%
*Streptococcus parasanguinis*	31	3	0/3	0.0%
*Comamonas testosteroni*	30	3	0/3	0.0%
*Actinomyces turicensis*	28	2	1/2	50.0%
*Prevotella buccae*	28	1	1/1	100.0%
*Clostridium innocuum*	27	1	0/1	0.0%
*Porphyrom. asaccharolytica*	27	1	1/1	100.0%
*Escherichia fergusonii*	25	1	0/1	0.0%
*Prevotella oris*	25	1	1/1	100.0%
*Parabacteroides distasonis*	24	21	4/21	19.0%
*Bacillus circulans*	23	1	0/1	0.0%
*Bacteroides thetaiotaomicron*	22	4	0/4	0.0%
*Citrobacter braakii*	22	1	1/1	100.0%
*Slackia exigua*	21	1	0/1	0.0%
*Eubacterium aerofaciens*	20	1	0/1	0.0%
*Solobacterium moorei	20	6	3/6	50.0%
*Hungatella hathewayi*	18	4	0/4	0.0%
*Bacteroides caccae*	16	4	1/4	25.0%
*Streptococcus pluranimalium*	12	1	0/1	0.0%
*Bacteroides stercoris*	10	1	0/1	0.0%
*Comamonas kerstersii*	10	2	1/2	50.0%
*Fusobact. gonidiaformans*	10	1	1/1	100.0%
*Eggerthia catenaformis*	9	1	1/1	100.0%
*Fusobacterium naviforme*	9	1	1/1	100.0%
*Eubacterium limosum	8	7	2/7	28.6%
*Collinsella aerofaciens*	7	4	1/4	25.0%
*Paeniclostridium sordellii*	6	1	0/1	0.0%
*Citrobacter youngae*	5	1	0/1	0.0%
*Bacteroides intestinalis*	4	2	0/2	0.0%
*Clostridium aldenense*	4	1	0/1	0.0%
*Porphyromonas somerae*	4	1	0/1	0.0%
*Bacteroides nordii*	3	2	0/2	0.0%
*Fusobacterium canifelinum*	3	1	0/1	0.0%
*Streptococcus massiliensis*	3	1	1/1	100.0%
*Bacillus simplex*	2	1	0/1	0.0%
*Clostridium citroniae*	2	1	1/1	100.0%
*Bacteroides cellulosilyticus*	1	1	0/1	0.0%
*Bacteroides salyersiae*	1	1	0/1	0.0%
*Bacteroides eggerthii*	1	1	0/1	0.0%
*Prevotella maculosa*	1	1	0/1	0.0%
*Escherichia coli (mucous)*	0	2	0/2	0.0%

Rare bacteria with elevated risk for complications marked with *.

If bacteria were detectable or not impacted the patient’s chance for complications: patients without detectable bacteria suffered complications in only 13.5% (28/207), while patients with bacterial growth had complications in 25.5% (95/372) (p<0.001).

To analyze the impact of rare pathogens on pediatric appendicitis, we compared patients with rare pathogens and those without rare pathogens with regards to complications: when no rare bacteria were present, patients suffered complications in 20.5% (38/185). This rate increased to 30.5% (57/187) when rare pathogens were identified (p<0.05). However, the impact of different bacteria varies greatly. Many rare bacteria have been detected less than 5 times in total. Many more have been detected together with complications only once. When we had more than 5 patients with a certain rare species that coincided in more than 20.5% with complications, we marked this species as “rare bacteria with elevated risk for complications”. In total, 8 different rare bacteria fulfilled these criteria (**Table 5**). They will be discussed later. There was no stringent correlation of rare pathogens to certain forms of appendicitis except for perforated appendicitis, where rare pathogens were found in 63.2%.

Following various publications, we identified standard calculated antibiotic regimes: ampicillin-sulbactam (Kambaroudis et al., 2010; Kronman et al., 2016), cefuroxime-metronidazole (Sauerland et al., 2010; Rollins et al., 2016), piperacillin-tazobactam (Fallon et al., 2013; Mazuski et al., 2017; Sartelli et al., 2017; Roque et al., 2019b) and imipenem (Kambaroudis et al., 2010; Mazuski et al., 2017; Sartelli et al., 2017; Roque et al., 2019b).

For 1330 different bacterial pathogens, we were able to obtain a resistogram. When testing resistances globally across all identified bacteria in all analyzed patients, only 21.6% were sensitive to ampicillin-sulbactam. The overall susceptibility was much higher against the combination of cefuroxime-metronidazole and piperacillin-tazobactam, reaching 72.3 and 78.9%. Finally, 88.4% of all found bacteria were susceptible to imipenem (**Supplementary Table 3**).

When considering all bacteria found in one specific patient, 91.4% of patients had at least one bacterium resistant to ampicillin-sulbactam, while only 30.9% of patients carried at least one bacterium resistant to imipenem. However, these proportions change when we include the patients without bacterial growth. In the total cohort of 579 patients, only 58.7% had bacteria resistant to ampicillin-sulbactam, 37.3% to cefuroxime + metronidazole, 31.4% to piperacillin-tazobactam, and 19.9% to imipenem (**Supplementary Table 4**).

We also examined rare and common bacteria separately concerning their resistances to ampicillin-sulbactam, cefuroxime with metronidazole, piperacillin-tazobactam, and imipenem: rare bacteria were more often resistant to ampicillin-sulbactam, but less often resistant to all other tested antibiotics. (**Supplementary Table 5**).

### Correlations of resistances with forms of appendicitis

To test the impact of antibiotic resistances on the course of pediatric appendicitis, we compared the rate of resistant bacteria in patients with different forms of appendicitis (**Table 6**). It is remarkable that patients with perforated appendicitis have higher chances of resistant bacterial growth against all tested antibiotics.

**Table 6:** Resistant bacteria in different forms of appendicitis.

Number of patients with at least 1 resistant pathogen against antibiotic	Catarrhal (n = 50)		Phlegmonous (n = 112)		Gangrenous (n = 74)		Perforated (n = 136)	
Ampicillin/Sulbactam	43	86.0%	92	82.1%	70	94.6%	135	99.3%
Cefuroxime/Metronidazole	25	50.0%	50	44.6%	44	59.5%	97	71.3%
Piperacillin/Tazobactam	22	44.0%	43	38.4%	34	45.9%	83	61.0%
Imipenem	11	22.0%	24	21.4%	26	35.1%	54	39.7%

### Correlation of resistances with complications

We also analyzed the presence of resistant bacteria in patients with and without complications. It is noteworthy that patients with complicated clinical courses have more frequently at least one resistant bacterium and consistently higher rates of resistant bacteria than those without complications (**Table 7**).

**Table 7:** Resistant bacteria and complications.

Number of patients with at least 1 resistant pathogen	Complication (n = 95)		No complication (n = 277)	
Ampicillin/Sulbactam	92	96.8%	248	89.5%
Cefuroxime/Metronidazole	77	81.1%	139	50.2%
Piperacillin/Tazobactam	59	62.1%	123	44.4%
Imipenem	38	40.0%	77	27.8%
Average rate of resistant bacteria in patients w...	with complications (n = 95)		without complications (n = 277)	
Ampicillin/Sulbactam	80.7%		71.9%	
Cefuroxime/Metronidazole	35.4%		22.9%	
Piperacillin/Tazobactam	24.0%		19.7%	
Imipenem	12.8%		10.2%	

### Correlation of bacterial growths with complications

The risk of complications was only 13.5% when no bacterial growth was seen. However, when the microbiological samples were unsterile, this rate increased to 25.6% (**Supplementary Table 6**).

Moreover, the rate of rare bacteria among all detected bacteria did not differ between patients suffering complications (23% rare bacteria) and those who did not suffer any complications (24% rare bacteria).

### Correlation of bacterial growth with forms of appendicitis and hospital stay

However, 46% of samples with at least one rare pathogen originated from perforated appendicitis but only 2.8% of sterile samples were taken in patients with perforation. Surprisingly, 25.1% of sterile samples were taken in catarrhal appendicitis when this rate was highest with 49.8% in phlegmonous appendicitis (**Supplementary Table 7**).

When comparing patients with sterile samples, to those with only common bacteria and those with rare bacteria, the latter two groups spent significantly more days in hospital. Patients with rare bacteria had the longest hospital stay (**Supplementary Figure 11**).

### Forms of appendicitis and bacterial growths as prognostic factors for complications

In a logistic regression model, only perforation was identified as statistically significant prognostic factor for complications with an odds ratio of 2.6 (95% CI 1.3 to 4.9) compared to catarrhal appendicitis. The elevated risk of rare pathogens for complications diminished after adjusting for kind of appendicitis (odds ratio 1.36; 95% CI 0.83 to 2.23, **Supplementary Table 8**).

## Discussion

### Strengths and weaknesses of this study

This study is the most comprehensive research on bacterial growths in different forms of pediatric appendicitis that gives insides on the role of common and rare bacteria as well as antibiotic susceptibility with regard to common calculated antibiotics, hospital stay and complications. However, it is a retrospective study. Furthermore, all included patients were treated in a single center reflecting the local situation. Still, antibiotic managements have to be prospectively evaluated and results might differ depending on local aspects. In addition, the exact culture methods for swabs and fluids were comparable but not 100% equal. Some methods were used less frequently on fluid samples. Therefore, the superiority of swabs over native fluid samples might be slightly overrated. However, the minimal difference on the frequency of culture techniques used cannot be held responsible for the tremendously better results of swabs.

There is no commonly agreed definition for “rare pathogens”. We here provide an approach based on the rarity of a clinical condition (appendicitis) in the presence of certain bacteria together with the scarcity of literature on this individual pathogen. However, we encourage and welcome future debate on this definition.

### How does our population compare to the literature?

This study investigated 619 patients of one center who underwent appendectomy. The 579 patients who met the inclusion criteria with an intraoperatively inflammatorily altered appendix represent the largest cohort considered for the study of appendectomies in this age group that we are aware of in the current literature. Regarding age and sex ratio, our population is consistent with the literature (Omling et al., 2019). The clinically suspected appendicitis was not confirmed intraoperatively in only 25 patients (n=25/579 = 3.9%). This low rate of false positive appendectomies is far below the 15% to 35% reported in the literature (Ohle et al., 2011; Brockman et al., 2013; Garcia et al., 2018). One reason may be that the initial conservative therapy, supportive measures in case of unclear findings, and repeated reevaluations by experienced surgeons are highly prioritized in our center. Intriguingly, this approach did not increase the rate of perforated appendicitis (24.5%) above numbers published in the literature for other tertiary centers (Smink et al., 2005). This finding could support reports suggesting a different entity of uncomplicated and complicated appendicitis compared to the classic progressive disease hypothesis.

The preferred surgical method in our clinic is the primary laparoscopic approach, either as a classic three port laparoscopy or as a single port approach (SILS). Only 4.8% of patients required a primary laparotomy, 95.2% were operated on laparoscopically. In 7.6%, an initial endoscopic procedure was converted intraoperatively to a laparotomy. Thus, a high rate of laparoscopic appendectomies (87.6%) is present in our population. Currently, the standard surgical method in Germany is still heterogeneous, and laparoscopic surgery is not yet established as the primary standard procedure in all hospitals because about 25% of appendectomies in Germany are still performed *via* laparotomy (Téoule et al., 2020) while worldwide, this rate is reported to be as high as 42% (Sartelli et al., 2018).

The average hospital stay of our patients was 7.9 days (2-41 days), and the median length of stay was six days. These numbers are not entirely due to medical needs but also reflect organizational standards and family needs in a center that serves a large rural area. In addition, no mortality occurred in our population when the literature still reports overall mortality of 0.09% up to 0.28% (Bhangu et al., 2015; Sartelli et al., 2018).

### What role do bacteria play in appendicitis?

In the majority of our cases, we were able to detect bacteria. Even in catarrhal appendicitis, bacteria were detectable in 49%. That underlines the role of bacterial migration in acute appendicitis, although it is still unclear if the bacterial infection is the reason for appendicitis or a secondary appearance. However, what we were able to show in the patient population studied, is the correlation of bacterial infection and the occurrence of complications in the further course. This is corroborated by the fact that the severity of clinical findings is positively related to the probability of positive bacterial detection. As the severity of the inflammatory change increases, so does the number of bacterial species detected. This is well explained by the further increasing permeability of the appendiceal wall, up to perforation. Also understandable is the increase in the frequency of complications with the detection of more bacterial pathogens. When patients with sterile swabs suffer complications in only 13.5%, they do so in 25.5% of cases with unsterile swabs (p<0.001). Another fact that supports the significance of bacterial infection in appendicitis is the different resistance patterns in patients with postoperative complications compared to those with an uneventful postoperative course.

### Do we really see transmigrated bacteria or are the bacteria in our samples due to iatrogenic contamination during laparoscopic handling?

We could not detect significant contamination with skin flora. Even in catarrhal appendicitis, the skin flora did not play a role in our population. However, the fact that more fluid samples that are commonly taken at the beginning of an operation prior to the excision of the appendix remained sterile supports the theory of intraoperative contamination of samples with intraluminal bacteria that might have been freed upon excision of the appendix.

However, the fact that the stage of the appendix inflammation directly correlates with the number of bacteria found argues against the contamination theory. Another fact that makes contamination of swabs in catarrhal and phlegmonous appendicitis unlikely is the high rate of unsterile swabs in open appendectomy since during open appendectomy, iatrogenic contamination of a swab with intraluminal bacteria is hardly imaginable. Future prospective studies should take intraoperative swabs at the beginning of the operation prior to the appendix excision with swabs suitable for laparoscopic approaches.

### Is it better to take swabs or to send in intraabdominal fluid?

Due to our retrospective analysis, we recommend Amies agar gel transported swabs. The tremendous rate of sterile fluid samples supports this recommendation. Swabs gave more unsterile results and a greater amount of different identified bacteria. Even when directly comparing fluid samples and swabs in patients who received both simultaneously, the swab outperformed the fluid sample. Finally, we would recommend a standardized procedure suitable for all patients. This standard can only be the swab since intraabdominal fluid is not always present in appendicitis.

### Are specific rare bacteria predictive of clinical complications?

Although in most appendicitis common bacterial pathogens can be found, one has to keep in mind that we detected rare bacteria in 32.3% (187/579). As mentioned above, authors repeatedly describe complications in appendicitis caused by rare bacteria (Paul and Patel, 2001; Almuzara et al., 2013; Forrester and Spain, 2014; Gardiner et al., 2015; Almuzara et al., 2017; Lopes et al., 2017; Legaria et al., 2020). This is most likely an example of a reporting bias since publications often arise from unusual complications while uncomplicated clinical courses remain underreported. The pathogenicity of rare bacteria is thus often overestimated. In our comprehensive approach, we could demonstrate an overrepresentation of rare bacteria in appendicitis with complications. Eight rare species were found to be associated to an above-average risk for complications with more than 2 patients affected.

*Solobacterium moorei* was detected in 6 patients of whom 3 suffered complications. This rare bacterium had thus the highest rate of complications in our population. It is an obligate anaerobic Gram-positive bacillus described mostly within the human oral cavity and human intestinal flora (Barrak et al., 2020). Recently several studies point to its role in oral infections. Being part of the tongue microbiota with beta-galactosidase activity potentially also producing volatile sulfur compounds, it is accused to cause halitosis (Barrak et al., 2020). Even though it is known to be an opportunistic pathogen in bloodstream and surgical site infections with excellent susceptibility to most antibiotics, there are some reports of *Solobacterium moorei* as being the only recovered bacteria in complicated infection (Alauzet et al., 2021). To our knowledge, our 6 patients of whom 3 suffered from complications, are the first appendicitis patients with *Solobacterium moorei* reported in literature.

*Gemella morbillorum* was detected in 7 patients of whom 3 suffered complications. It has been first described in 1917 as *Streptococcum morbillorum* and is part of the normal flora of human oropharynx, genitourinary system, and gastrointestinal system (Romero-Velez et al., 2020). There are case reports of *G. morbillorum* causing necrositing fasciitis of the torso, thoracic aortic aneurysm, and endocarditis (Ural et al., 2014; Romero-Velez et al., 2020; Said and Tirthani, 2021). To our knowledge, our 7 patients of whom 3 suffered from complications, are the first appendicitis patients with *G.morbillorum* reported in literature.

*Bacteroides uniformis* was detected in 11 patients of whom 4 suffered complications. It is part of the human gut microbia (Grondin et al., 2022) and is thought to have anti-obesity effects. Although being described as a pathogen in human appendices more than 40 years ago, *B. uniformis* is very rarely mentioned in literature in regards of appendicitis (Elhag et al., 1986).

*Eggerthella lenta* was detected in 6 patients of whom 2 suffered complications. It is anaerobic, non-sporulating, Gram positive and part of the normal human microflora (Jiang et al., 2021). *E. lenta* has been described to cause appendicitis (Jiang et al., 2020; Jiang et al., 2021).

*Parvimonas micra* was detected in 12 patients of whom 4 had complications. It is a fastidious, anaerobic, Gram−positive coccus that is found in healthy human oral and gastrointestinal flora (Xu et al., 2020). It is described as a rare cause of spondylodiscitis (Yoo et al., 2019). Changes in the abundance of *P. micra* have been described in children with complex appendicitis (Durovic et al., 2020). However, 8 of our 12 patients with *P. micra* had an uneventful clinical course without complications.

*Eubacterium limosum* was detected in 7 patients of whom 4 had complications. It is a Gram-positive, methanol-utilizing aceto-gen (Flaiz et al., 2021). *E. limosum* is a human gut symbiont (Ellenbogen et al., 2021). To our knowledge, our 7 patients of whom 3 suffered from complications, are the first appendicitis patients with *E. limosum* reported in literature.

*Bacteroides ovatus* was detected in 30 patients of whom 7 had complications. It is a gram-negative human gut bacteria able to suppress inflammation in the gastrointestinal tract (Fultz et al., 2021). There are few reports for *B. ovatus* being isolated in patients after appendectomy (Tocchioni et al., 2016; Fuse et al., 2022; Ward et al., 2022).

*Bilophila wadsworthia* was detected in 54 patients of whom 16 had complications, thus having the highest total number of associated complications. It is a Gram-negative sulfite-reducing human gut bacillus (Natividad et al., 2018). Recently several studies point to its role in the human gut microbiome (David et al., 2014). Though it is well known to be associated with appendicitis and colitis (Burrichter et al., 2021), many other infections like scrotal abscess, mandibular osteomyelitis or bacteremia have been described in relation to *B. wadsworthia* (Finegold et al., 1992; Kasten et al., 1992).

However, of 63 rare bacteria in our study, 32 were detected in patients who recovered without any problems. 6 rare pathogens were detected in our population two or more times without any associated complications. Among them were *Bacteroides thetaiotaomicron*, that could be found in 4 patient of whom no one suffered complications. This bacteria is seldom reported in literature. It is a Gram-negative, anaerobic gut bacteria, which is considered a high efficient degrader of polysaccharides and a potential probiotic. We were able to find 2 reports of wound- (Agarwal et al., 2014) and knee joint infection (Brandariz-Núñez and Gálvez-López, 2021) caused by this species. The wound infection occurred in a chronically ill patient with disseminated myeloma, and the knee infection occurred in a previously healthy young man after several surgical procedures on the knee. However, we consider this pathogen to be opportunistic and, according to our data, without great clinical relevance.

*Hungatella hathewayi* was also associated with no complications and could be found in 4 patients. This bacterium is Gram-negative, anaerobic species is reported in connection with the development of eczema in early childhood (Chan et al., 2021). We could find two reports of septicemia in the setting of perforated appendicitis with this pathogen (Woo et al., 2004; Randazzo et al., 2015). Thus, this bacterium appears to have clinical relevance. Based on the antibiotic therapy administered, this bacterium seems to have been adequately treated. Maybe, we could not find any complications in our patient population, due to the low complication rate and the small size of our sample.

*Streptococcus parasanguinis* was detected in 3 patients without any reported complications. This Gram-positive bacterium is usually found in the mouth, where it is a plaque-forming agent. It also plays a role in subacute endocarditis, especially after dental surgery, and causes bone infection of the periodontium (Chen et al., 2020). Additionally, we could find two reports of osteomyelitis of the spine or skull base with *Strep. parasanguinis* (Valanejad and Hill, 2020; Kim et al., 2021), in both cases as combined infection with other pathogens. According to the current state of the literature, complications with this pathogen appear to be limited to older, previously ill or immunocompromised patients.

*Comamonas testosteronii* was found in 3 patients of whom no one suffered complications. That is a very interesting, because this Gram-negative, wide spread environmental bacteria is often reported in association with human infection and appendicitis (Gul et al., 2007; Khalki et al., 2016). Remarkable is the fact of the high reported frequency of this species in perforated appendicitis in rather young patients (Tiwari and Nanda, 2019; Miloudi et al., 2020). Infections with this bacterium must be considered in view of the current literature and the now numerous reports in younger patients. However, this pathogen has shown a good response to standard antibiotics. Also, the possibility of a broad resistance to antibiotics has already been discussed and should be considered (Miloudi et al., 2020).

*Bacteroides intestinalis* was found in 2 patients without complications. This bacteria has not been reported with pathological findings, yet. It is considered as a useful commensal of the human gut with the ability to degrade dietary fiber with even health benefits (Yasuma et al., 2021). Of course, this bacteria has only been detected in our population together with other bacteria.

*Bacteroides nordii* was found in 2 patients without any complications. This Gram negative, anaerobic bacterium is a naturally occurring component of the microbiome. It has been isolated previously from abdominal swabs, e.g. in perforated appendicitis, but always in mixed cultures (Song et al., 2004). There has been no evidence of manifest infections by this bacterium to date, nor has there been any evidence of it as a pathogen in blood culture. Therefore a low virulence is considered.

Since complications are not frequent overall, they are even less often observed with rare bacteria. The elevated risk of rare pathogens on complications found in univariate analysis became insignificant after adjusting for the different forms of appendicitis. Additionally, the rare bacteria detected in our study population were always found together with other bacterial growth. Therefore it is not possible to attribute the eventual complications to the rare bacteria when other, common bacteria were also present. In summary, it was not possible to get statistically significant data on the relative risk profile of certain rare bacteria in pediatric appendicitis in our study population. Maybe if more comprehensive reports on bacteria in appendicitis were published, meta-analyses could elicit specific risk profiles of different bacteria in the future.

### Is perioperative antibiotic prophylaxis useful?

Although our retrospective study did not intend to evaluate the benefit of perioperative antibiotics clinically, our data support its routine use. Many authors would argue that no antibiotics are needed in appendectomy when dealing with uncomplicated appendicitis (Kizilcan et al., 1992). On the other hand, authors argue that uncomplicated appendicitis can be treated with antibiotics only (Di Saverio et al., 2020). Thus, bacterial infections seem to play a role even in uncomplicated appendicitis. Our data show that intraperitoneal bacteria is present in even more than 50% of catarrhal and phlegmonous appendicitis. Since past research has demonstrated that surgeons tend to underestimate the degree of inflammation in laparoscopic appendectomy (Holloway et al., 2020), it does not make much sense to spare antibiotic prophylaxis for intraoperatively diagnosed gangrenous appendicitis. It seems more sensible to administer perioperative antibiotics 30 minutes prior to incision while reserving prolonged therapy for complicated disease (Daskalakis et al., 2014; Gorter et al., 2016; Di Saverio et al., 2020).

### Which calculated antibiotics should be used?

Given our findings, imipenem would undoubtedly be the best calculated antibiotic. However, since imipenem is considered a reserve antibiotic (Roque et al., 2019a), piperacillin-tazobactam should be the calculated substance of choice. Since most severe complications were noticed in patients whose rate of bacteria resistant to piperacillin-tazobactam was higher, imipenem remains a good choice for calculated escalation of antibiotic management. Only 11.6% of all bacteria found in this study were resistant to imipenem. Even in perforated appendicitis, imipenem-resistant bacteria were only found in 12.0%. When looking at patients with severe complications, only 13.0% of their intraoperatively found bacteria were resistant to imipenem. This data only reflects the situation in our region and is subject to changes with time. Also, different antibiotics should be evaluated in a prospective trial.

## Conclusion

Bacteria play an important role in all forms of appendicitis and, most of all, in its complications. Therefore, standard bacterial swabs should be taken intraoperatively from the appendix before its excision. Based on the detected microbiomes in this study, for pre-operative prophylaxis and, if needed, for antibiotic treatment, piperacillin-tazobactam would be a reasonable first choice. Imipenem can cover up to 88% of expected bacteria when calculated escalation of antibiotic treatment is needed. This strategy should be evaluated in larger, prospective studies. Future studies are also required to elicit certain rare bacteria’s roles and pathomechanisms when their pathogenicity was overrated due to the apparent publication bias in case reports that can be overcome by comprehensive approaches as we have presented here.

## Data availability statement

The data analyzed in this study is subject to the following licenses/restrictions: Anonymized extracted data is available upon reasonable request at the corresponding author. Requests to access these datasets should be directed to jurek.schultz@uniklinikum-dresden.de.

## Ethics statement

The studies involving human participants were reviewed and approved by Institutional Review Board Technical University Dresden Medical Faculty Fetscherstraße 74 01307 Dresden IORG0001076, IRB00001473. Written informed consent from the participants’ legal guardian/next of kin was not required to participate in this study in accordance with the national legislation and the institutional requirements.

## Author contributions

JF: literature research, data extraction, data analysis, statistics, preparation of figures, preparation of manuscript, selection of references, writing of manuscript, reviewing and proof reading. BG: data analysis, statistics, preparation of figures, literature research and selection of references, reviewing and proof reading, submitting the manuscript. AR: data extraction, data analysis, preparation of figures, preparation of manuscript, literature research and selection of references, writing of manuscript, reviewing and proof reading. EW: data analysis, preparation of figures, literature research and selection of references, preparation of manuscript, writing of manuscript, English language editing, reviewing and proof reading. ET: data analysis, statistics, preparation of figures, reviewing and proof reading. SP: conceptualization of study, planning of study, data analysis, reviewing and proof reading. GF: conceptualization of study, planning of study, literature research and selection of references, reviewing and proof reading. JS: conceptualization of study, planning of study, data extraction, data analysis, statistics, preparation of figures, preparation of manuscript, literature research and selection of references, writing of manuscript, English language editing, reviewing and proof reading, submitting the manuscript. All authors contributed to the article and approved the submitted version.

## Supplementary material

Supplementary Table 1 Comparison of Patients with swab only, fluid sample only, and both.

Supplementary Table 2 sterile and unsterile results with regards to mode of appendectomy.

Supplementary Table 3 Antibiotics susceptibility of identified bacteria.

Supplementary Table 4 Patients with resistant bacteria.

Supplementary Table 5 Resistances of rare and common bacteria to standard antibiotics.

Supplementary Table 6 Sterile, unsterile, and unsterile samples with rare pathogens in patients with and without complications.

Supplementary Table 7 Different forms of appendicitis when no, only common or rare bacteria were found.

Supplementary Table 8 Logistic regression for complications in different forms of appendicitis and with only common bacteria, no bacteria, or rare bacteria, when adjusted for form of appendicitis (CI = confidence interval).

Supplementary Figure 1 Laboratory processing of swabs and fluids.

Supplementary Figure 2 Length of in-patient treatment for appendicitis with appendectomy.

Supplementary Figure 3 Top-20-profiles of bacteria identified in fluids **(A)** and swabs **(B)**.

Supplementary Figure 4 Top 10 bacteria in catarrhal appendicitis.

Supplementary Figure 5 Top 10 bacterial pathogens in phlegmonous appendicitis.

Supplementary Figure 6 Top 10 bacterial pathogens in gangrenous appendicitis.

Supplementary Figure 7 Top 10 bacterial pathogens in perforated appendicitis.

Supplementary Figure 8 Number of identified bacteria per patient (sterile samples excluded).

Supplementary Figure 9 Top-6 genus of all identified bacteria.

Supplementary Figure 10 Amount of patients in whom certain genera were identified.

Supplementary Figure 11 hospital stay in the presence of sterile, only common bacteria or at least one rare pathogen (p<0.001).
