# Supplementary material for: Bacterial pathogens in pediatric appendicitis: a comprehensive retrospective study
Source: Front Cell Infect Microbiol. 2023 May 9;13:1027769. doi: 10.3389/fcimb.2023.1027769 (PMC10205019; doi:10.3389/fcimb.2023.1027769)
Supplement: Supplementary Table 1 — Comparison of Patients with swab only, fluid sample only, and both. [file Table_1.pdf]

	Swab only n = 387		Fluid sample only n = 82		Both n = 110	
**Age**						
average	10.1		11.2		10.6	
SD	3.87		3.52		3.62	
**Sex**						
male	218	56.3%	32	39.0%	58	52.7%
female	169	43.7%	50	61.0%	52	47.3%
**Form**						
catarrhal	60	15.5%	19	23.2%	23	20.9%
phlegmonous	144	37.2%	30	36.6%	41	37.3%
gangrenous	79	20.4%	19	23.2%	22	20.0%
perforated	104	26.9%	14	17.1%	24	21.8%
**OP-technique**						
laparoscopic	327	84.5%	80	97.6%	102	92.7%
open	25	6.5%	0	0.0%	3	2.7%
converted	35	9.0%	2	2.4%	5	4.5%
**Complications**						
with complications	80	20.7%	16	19.5%	28	25.5%
without complications	307	79.3%	66	80.5%	82	74.5%

Supplementary Table 1: Comparison of Patients with swab only, fluid sample only, and both.
