# Supplementary material for: Bacterial pathogens in pediatric appendicitis: a comprehensive retrospective study
Source: Front Cell Infect Microbiol. 2023 May 9;13:1027769. doi: 10.3389/fcimb.2023.1027769 (PMC10205019; doi:10.3389/fcimb.2023.1027769)
Supplement: Supplementary Table 2 — sterile and unsterile results with regards to mode of appendectomy. [file Table_2.pdf]

Total (n = 579)	Laparoscopic (n = 509)		Open (n = 28)		Converted (n = 42)	
sterile (n = 207)	202	39.7%	3	10.7%	2	4.8%
bacteria (n = 372)	307	60.3%	25	89.3%	40	95.2%

Supplementary Table 2: sterile and unsterile results with regards to mode of appendectomy.
