# Supplementary material for: Bacterial pathogens in pediatric appendicitis: a comprehensive retrospective study
Source: Front Cell Infect Microbiol. 2023 May 9;13:1027769. doi: 10.3389/fcimb.2023.1027769 (PMC10205019; doi:10.3389/fcimb.2023.1027769)
Supplement: Supplementary Table 3 — Antibiotics susceptibility of identified bacteria. [file Table_3.pdf]

Total (n = 1330)	Ampicillin/ Sulbactam		Cefuroxime/ Metronidazole		Piperacillin/ Tazobactam		Imipenem	
resistant	1043	78.4%	369	27.7%	281	21.1%	154	11.6%
sensitive	287	21.6%	961	72.3%	1049	78.9%	1176	88.4%

Supplementary table 3: Antibiotics susceptibility of identified bacteria.
