# Supplementary material for: Bacterial pathogens in pediatric appendicitis: a comprehensive retrospective study
Source: Front Cell Infect Microbiol. 2023 May 9;13:1027769. doi: 10.3389/fcimb.2023.1027769 (PMC10205019; doi:10.3389/fcimb.2023.1027769)
Supplement: Supplementary Table 4 — Patients with resistant bacteria. [file Table_4.pdf]

Patients with non-sterile results (n = 372)	Ampicillin/ Sulbactam		Cefuroxime/ Metronidazole		Piperacillin/ Tazobactam		Imipenem	
average proportion of resistant bacteria in a patient	74.1%		26.1%		20.8%		10.9%	
patients with at least one resistant bacteria	340	91.4%	216	58.1%	182	48.9%	115	30.9%

Supplementary table 4: Patients with resistant bacteria.
