# Supplementary material for: Bacterial pathogens in pediatric appendicitis: a comprehensive retrospective study
Source: Front Cell Infect Microbiol. 2023 May 9;13:1027769. doi: 10.3389/fcimb.2023.1027769 (PMC10205019; doi:10.3389/fcimb.2023.1027769)
Supplement: Supplementary Table 5 — Resistances of rare and common bacteria to standard antibiotics. [file Table_5.pdf]

Resistant against:	Rare pathogens (n = 312)		Common pathogens (n = 1018)		Total (n = 1330)	
Ampicillin/Sulbactam	277	88.8%	766	75.2%	1043	78.4%
Cefuroxime/Metronidazole	68	21.8%	301	29.6%	369	27.7%
Piperacillin/Tazobactam	44	14.1%	237	23.3%	281	21.1%
Imipenem	13	4.2%	141	13.9%	154	11.6%

Supplementary table 5: Resistances of rare and common bacteria to standard antibiotics.
