# Supplementary material for: Bacterial pathogens in pediatric appendicitis: a comprehensive retrospective study
Source: Front Cell Infect Microbiol. 2023 May 9;13:1027769. doi: 10.3389/fcimb.2023.1027769 (PMC10205019; doi:10.3389/fcimb.2023.1027769)
Supplement: Supplementary Table 6 — Sterile, unsterile, and unsterile samples with rare pathogens in patients with and without complications. [file Table_6.pdf]

Total (n = 579)	Sterile (n = 207)		Number of patients with only common pathogens (n = 185)		Number of patients with at least 1 rare pathogen (n = 187)	
with complications (n = 123)	28	13.5%	38	20.5%	57	30.5%
without complications (n = 456)	179	86.5%	147	79.5%	130	69.5%
p-value	< 0.001					

Supplementary table 6: Sterile, unsterile, and unsterile samples with rare pathogens in patients with and without complications.
