# Supplementary material for: Bacterial pathogens in pediatric appendicitis: a comprehensive retrospective study
Source: Front Cell Infect Microbiol. 2023 May 9;13:1027769. doi: 10.3389/fcimb.2023.1027769 (PMC10205019; doi:10.3389/fcimb.2023.1027769)
Supplement: Supplementary Table 7 — Different forms of appendicitis when no, only common or rare bacteria were found. [file Table_7.pdf]

	No bacteria detected /sterile (n = 207 patients)		Only common bacteria (n = 185 patients)		At least 1 rare pathogen (n = 187 patients)	
catarrhal (n = 102)	52	25.1%	23	12.4%	27	14.4%
phlemonous (n = 215)	103	49.8%	71	38.4%	41	21.9%
gangrenous (n = 120)	46	22.2%	41	22.1%	33	17.6%
perforated (n = 142)	6	2.8%	50	27.0%	86	46.0%
p-value	< 0.001					

Supplementary table 7: Different forms of appendicitis when no, only common or rare bacteria were found.
