# Supplementary material for: Bacterial pathogens in pediatric appendicitis: a comprehensive retrospective study
Source: Front Cell Infect Microbiol. 2023 May 9;13:1027769. doi: 10.3389/fcimb.2023.1027769 (PMC10205019; doi:10.3389/fcimb.2023.1027769)
Supplement: Supplementary Table 8 — Logistic regression for complications in different forms of appendicitis and with only common bacteria, no bacteria, or rare bacteria, when adjusted for form of appendicitis (CI = confidence interval). [file Table_8.pdf]

Prognostic factor	Odds ratio		
	estimate	95% CI	p-value
Appendicitis			<0.001
catarrhal	reference		
phlegmonous	0.63	0.32 – 1.21	
gangrenous	1.14	0.58 – 2.26	
perforated	2.55	1.33 – 4.89	
Bacteria			0.225
common	reference		
no bacteria detected	0.84	0.48 – 1.49	
at least one rare pathogen	1.36	0.83 – 2.23	

Supplementary table 8: Logistic regression for complications in different forms of appendicitis and with only common bacteria, no bacteria, or rare bacteria, when adjusted for form of appendicitis (CI = confidence interval).
