# Supplementary figures and images for: Bacterial pathogens in pediatric appendicitis: a comprehensive retrospective study
Source: Front Cell Infect Microbiol. 2023 May 9;13:1027769. doi: 10.3389/fcimb.2023.1027769 (PMC10205019; doi:10.3389/fcimb.2023.1027769)

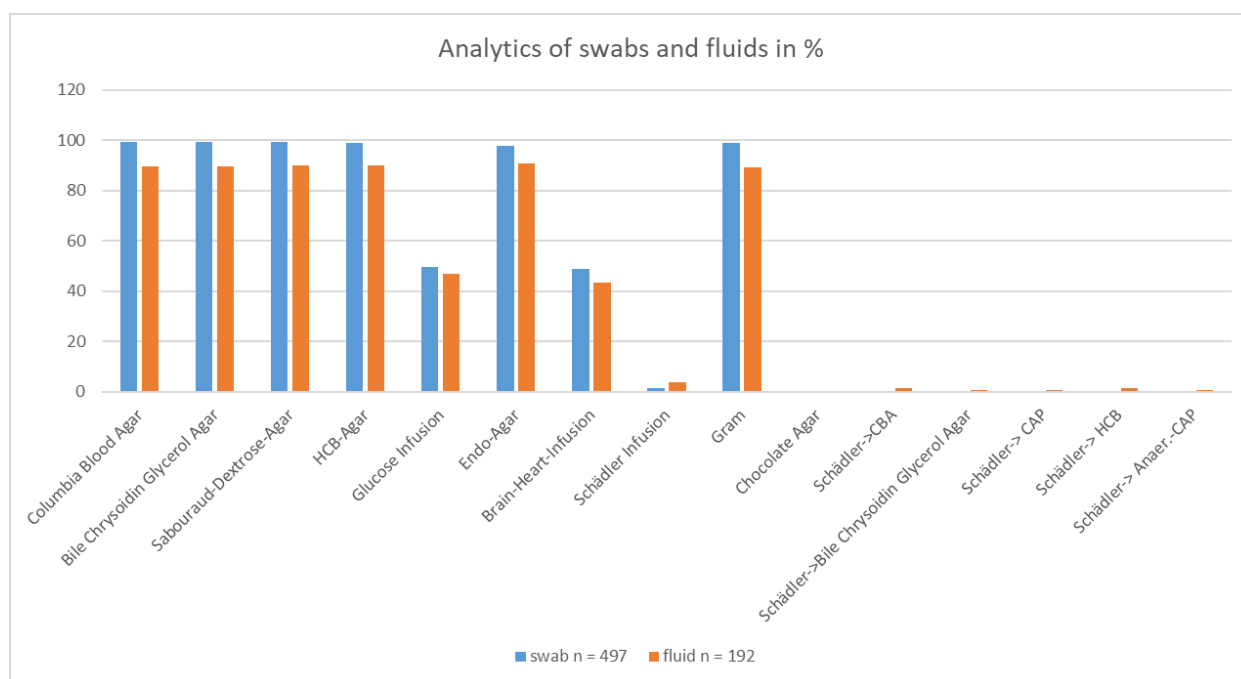

Supplementary figure 1: Laboratory processing of swabs and fluids.

Supplement: Supplementary Figure 1 — Laboratory processing of swabs and fluids. [file Image_1.pdf]

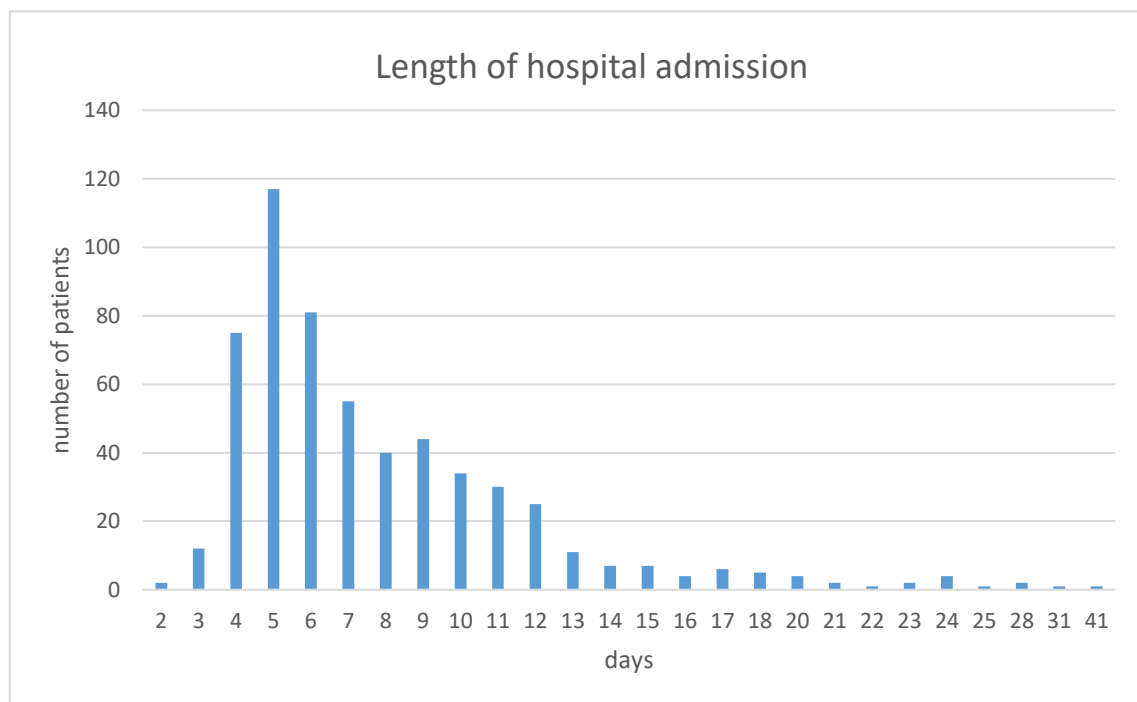

Supplementary Figure 2: Length of in-patient treatment for appendicitis with appendectomy.

Supplement: Supplementary Figure 2 — Length of in-patient treatment for appendicitis with appendectomy. [file Image_2.pdf]

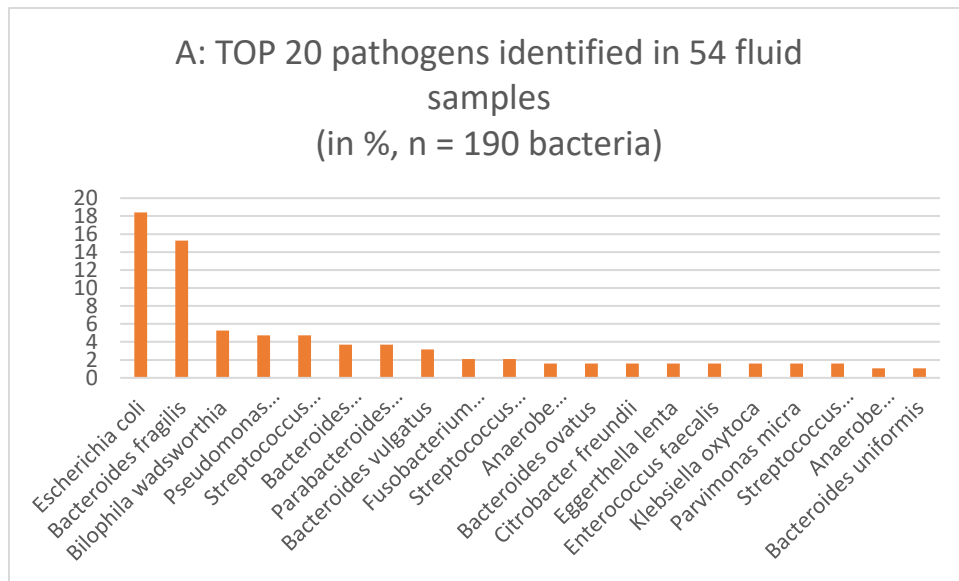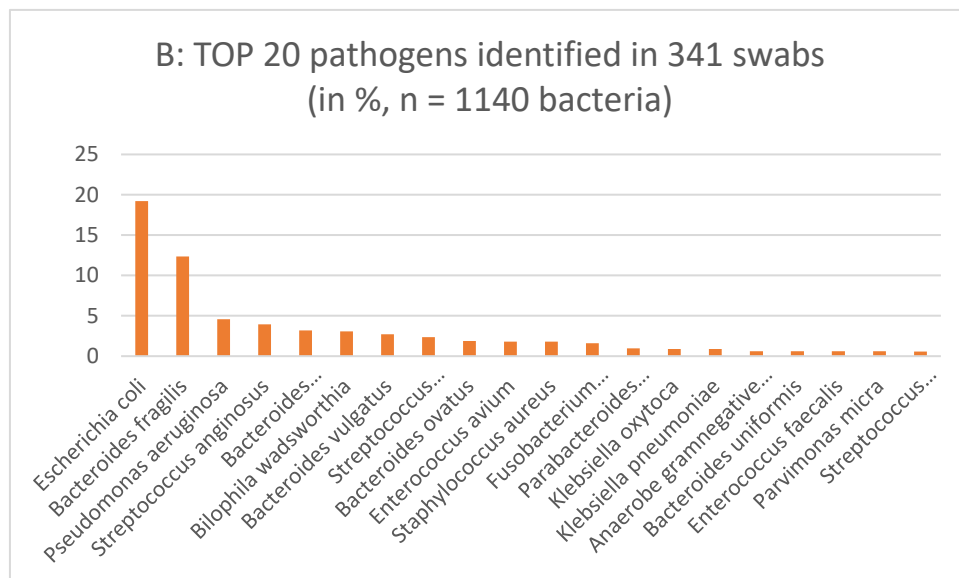

Supplementary Figure 3: Top-20-profiles of bacteria identified in fluids (A) and swabs (B).

Supplement: Supplementary Figure 3 — Top-20-profiles of bacteria identified in fluids (A) and swabs (B). [file Image_3.pdf]

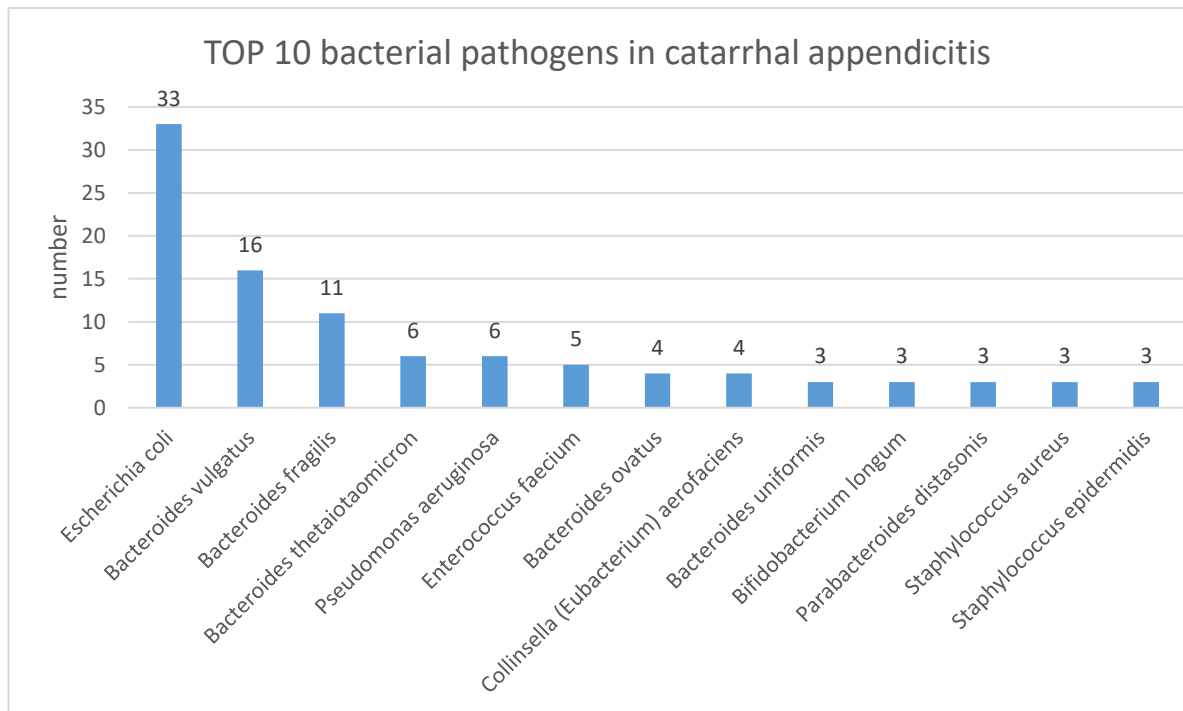

Supplementary Figure 4: Top 10 bacteria in catarrhal appendicitis.

Supplement: Supplementary Figure 4 — Top 10 bacteria in catarrhal appendicitis. [file Image_4.pdf]

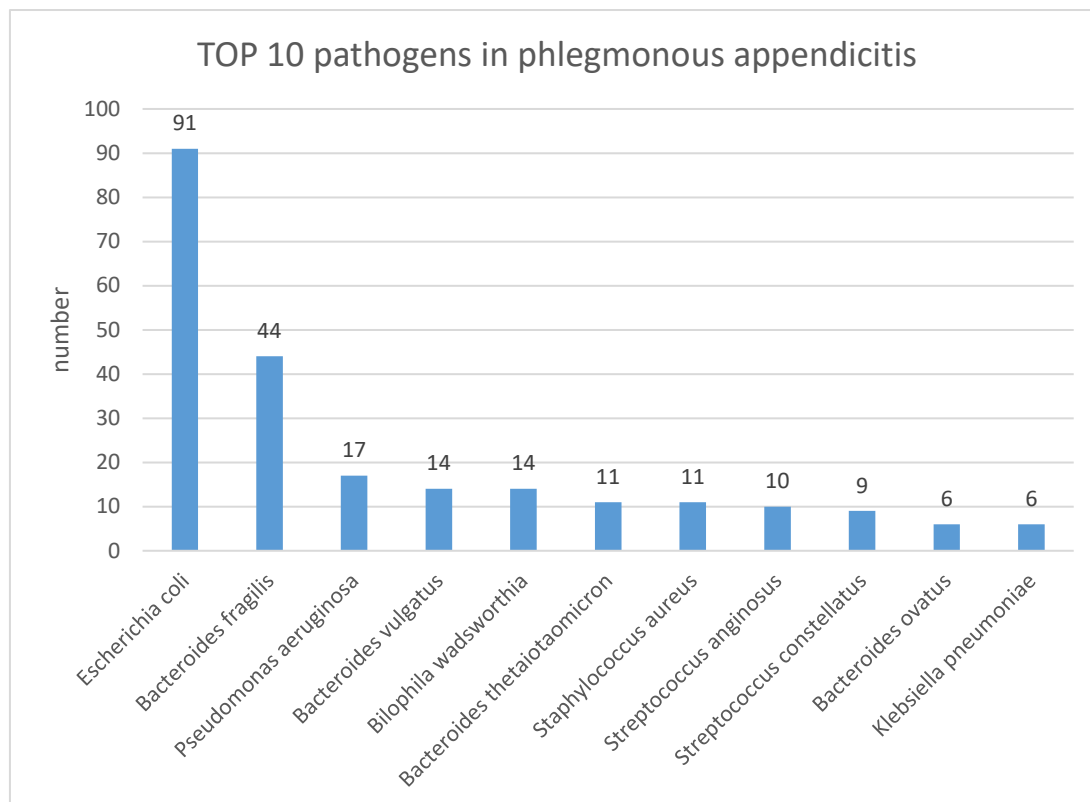

Supplementary Figure 5: Top 10 bacterial pathogens in phlegmonous appendicitis.

Supplement: Supplementary Figure 5 — Top 10 bacterial pathogens in phlegmonous appendicitis. [file Image_5.pdf]

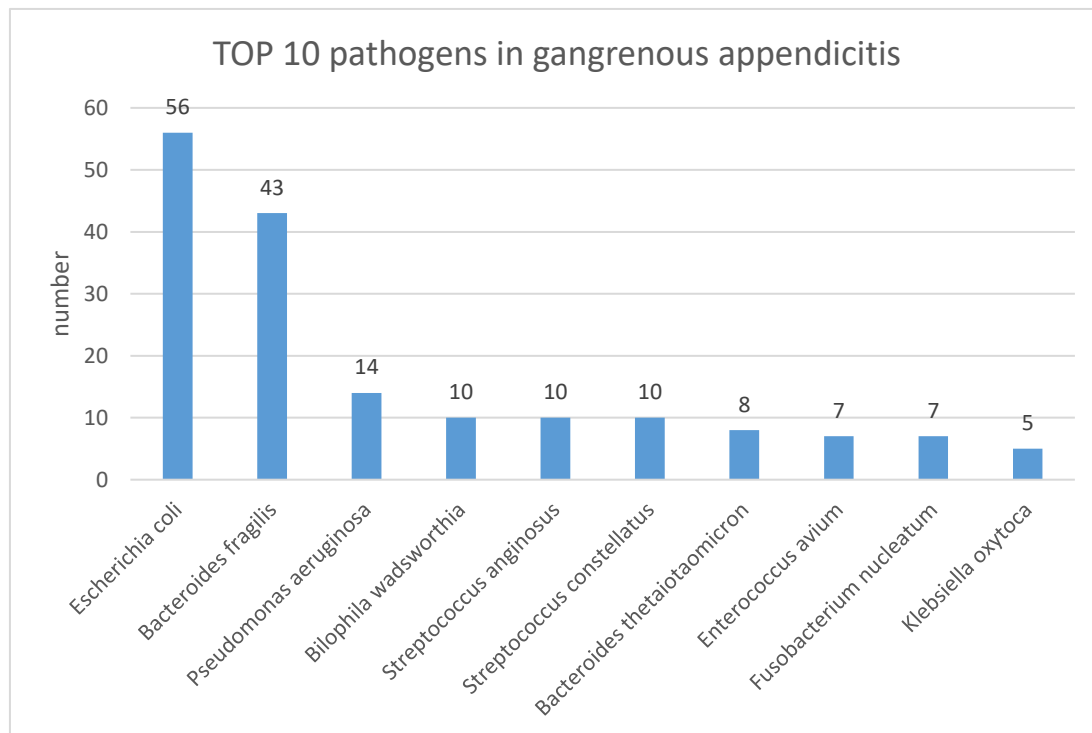

Supplementary Figure 6: Top 10 bacterial pathogens in gangrenous appendicitis.

Supplement: Supplementary Figure 6 — Top 10 bacterial pathogens in gangrenous appendicitis. [file Image_6.pdf]

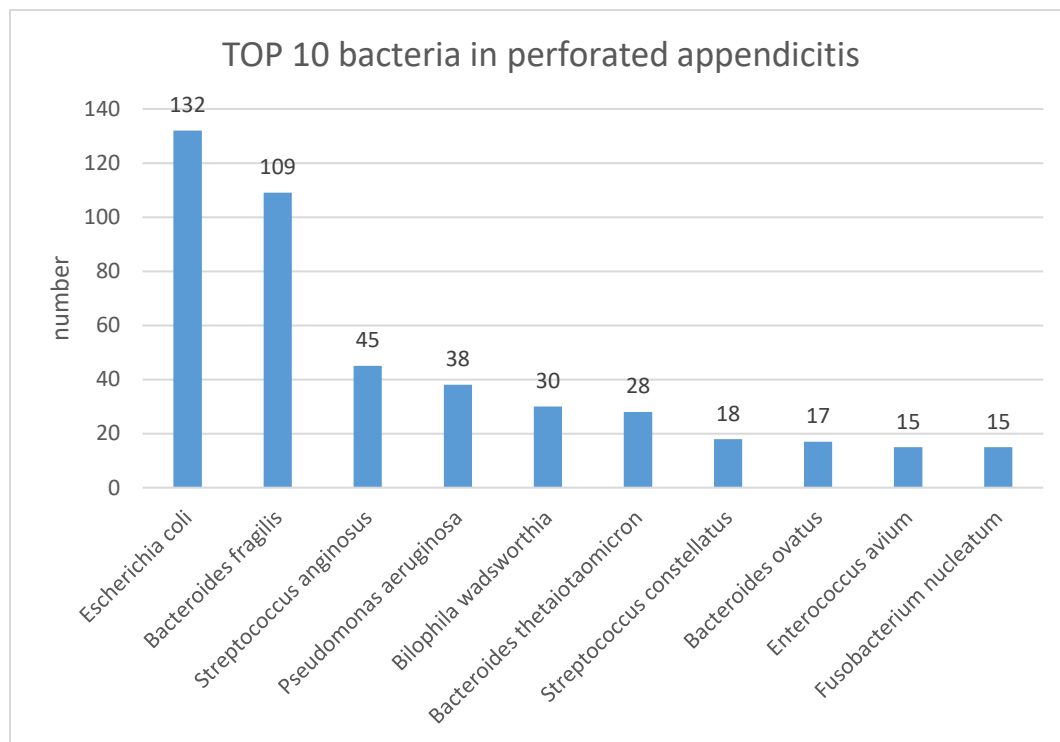

Supplementary Figure 7: Top 10 bacterial pathogens in perforated appendicitis.

Supplement: Supplementary Figure 7 — Top 10 bacterial pathogens in perforated appendicitis. [file Image_7.pdf]

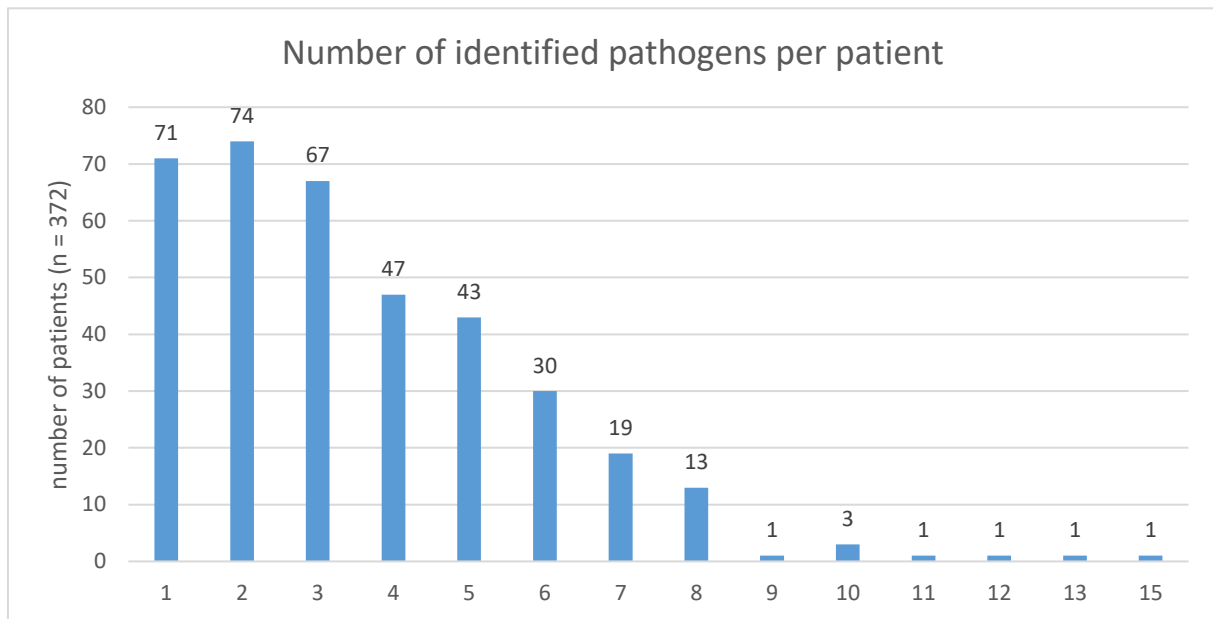

Supplementary Figure 8: Number of identified bacteria per patient (sterile samples excluded).

Supplement: Supplementary Figure 8 — Number of identified bacteria per patient (sterile samples excluded). [file Image_8.pdf]

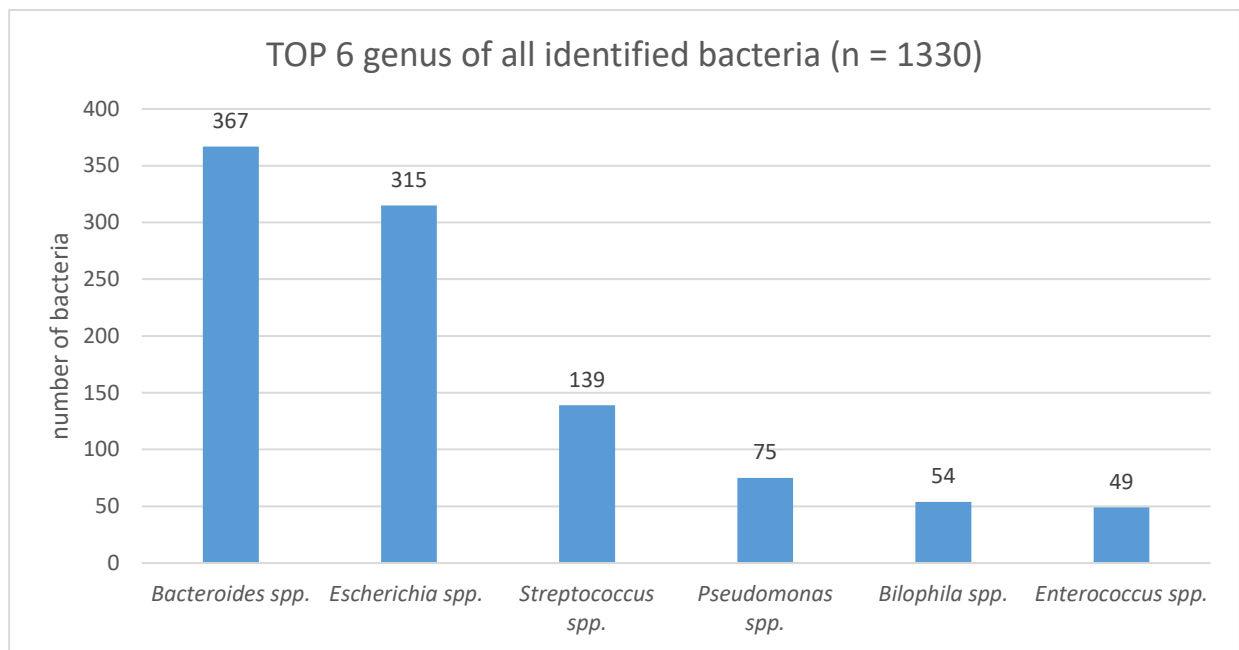

Supplementary Figure 9: Top-6 genus of all identified bacteria.

Supplement: Supplementary Figure 9 — Top-6 genus of all identified bacteria. [file Image_9.pdf]

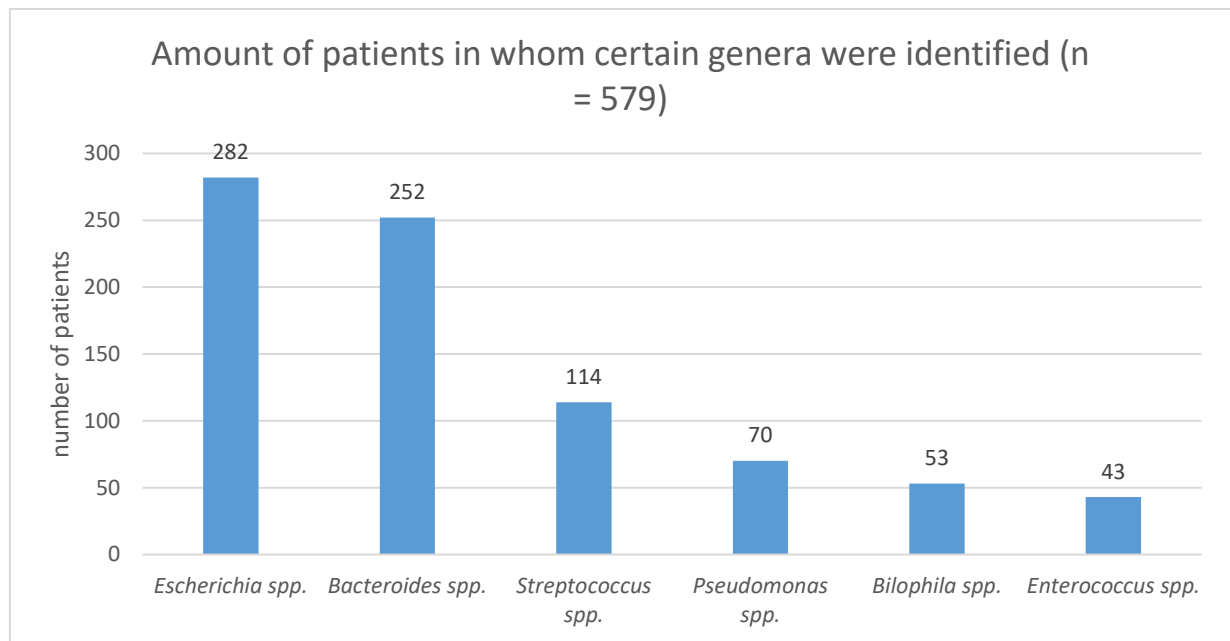

Supplementary Figure 10: Amount of patients in whom certain genera were identified.

Supplement: Supplementary Figure 10 — Amount of patients in whom certain genera were identified. [file Image_10.pdf]
