# Supplementary material for: Bacterial pathogens in pediatric appendicitis: a comprehensive retrospective study
Source: Front Cell Infect Microbiol. 2023 May 9;13:1027769. doi: 10.3389/fcimb.2023.1027769 (PMC10205019; doi:10.3389/fcimb.2023.1027769)
Supplement: Supplementary Figure 11 — hospital stay in the presence of sterile, only common bacteria or at least one rare pathogen (p<0.001). [file Image_11.pdf]

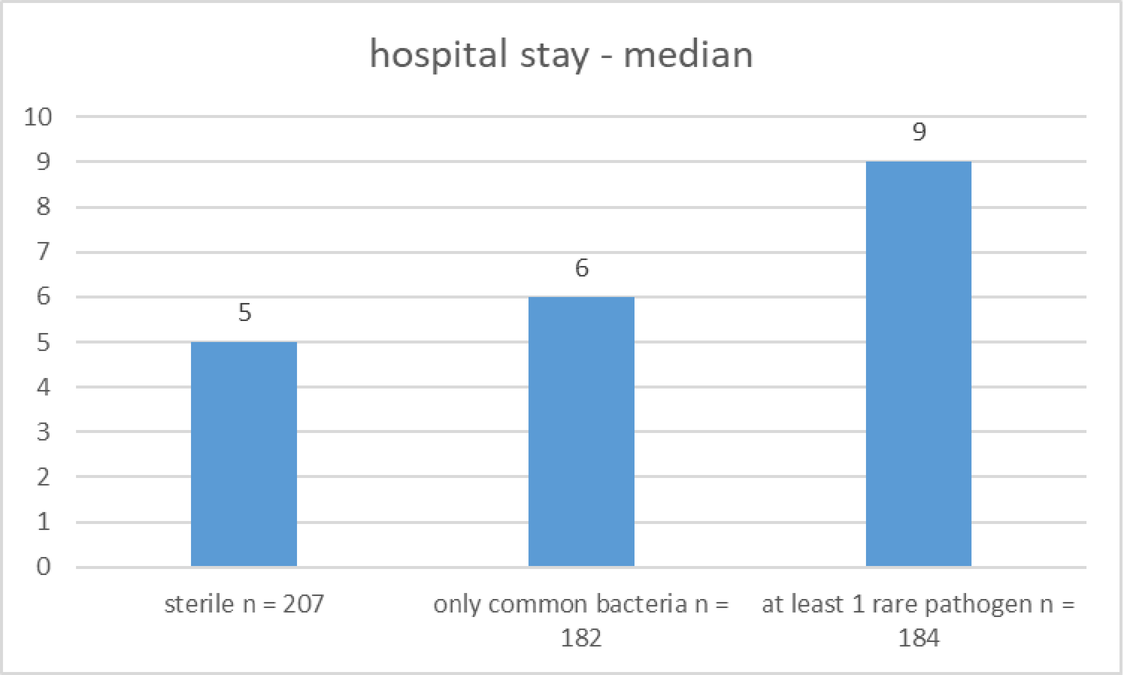

Supplementary figure 11: hospital stay in the presence of sterile, only common bacteria or at least one rare pathogen (p<0.001).
